# Extract of *Aloe vera* (*Aloe barbadensis* Miller) Enhances the Growth, Protein Contents, and Gastrosomatic Index (GaSI) of Common Carp *Cyprinus carpio*

**DOI:** 10.1155/2021/8029413

**Published:** 2021-03-04

**Authors:** Munu Khanal, Sirjana Lamichhane, Ajaya Bhattarai, Babita Labh Kayastha, Shyam Narayan Labh

**Affiliations:** ^1^Department of Zoology, Amrit Campus, Tribhuvan University, Kathmandu, Nepal; ^2^Department of Chemistry, M. M. A. M. Campus, Tribhuvan University, Biratnagar, Nepal; ^3^Nepal Aquaculture Society, Vinayak Marg Sinamagal-9, Kathmandu, Nepal

## Abstract

Aquaculture is a growing agribusiness, and large-scale microbial infection frequently leads to considerable economic losses, as there are very few approved drugs available to counteract such a problem. *Aloe vera* is a natural therapeutic plant with biological activities, for example, antimicrobial, anticancer, mitigating, and immunomodulatory properties. Thus, an experiment was conducted to understand the effects of dietary *Aloe vera* on survival, growth performance, protein utilization, gastrosomatic index, and gut histomorphological studies in common carp in laboratory conditions. *Aloe vera* leaves were collected, washed, shade-dried, and homogenized to get a filtrate, which was extracted in ethanol (70%) using the Soxhlet apparatus. The carp with an average weight of 1.73 ± 0.14 g was divided randomly into 5 treatments (control and other 4 treated groups) with the extract of 0.0%, 0.2%, 0.4%, 0.8%, and 1.6% in the diet with 3 replicates and the stocking density was 25 fish per each replicate. Sampling was done on the 30th, 60th, and 90th days, and the results show that percent weight gain, specific growth rate, protein efficiency ratio, and feed conversion efficiency were significantly high in the treated diet compared to the control (0.0%). Gastrosomatic index, condition factors, and gut histology found better performance in common carp group fed 0.4% and 0.8% of *Aloe vera* after 90 days. In conclusion, it was found that *Aloe vera* extract between the amount of 0.4% and 0.8% is better for fish growth for common carp.

## 1. Introduction

Immunostimulants are of unfamiliar interest in medical care and have gotten quite possibly the most dynamic zones of applied clinical exploration [1]. Immunostimulants are dietary added substances that upgrade guard instruments and increment protection from explicit microbes.

Moreover, practices in the use of immunostimulants as a choice in contrast to drugs, substances, and antibiotics are environment friendly and accessible in different places of the world. It improves the intrinsic (or vague) immune response, which has a more conspicuous part in fish immunity [2, 3]. In this way, the utilization of immunostimulants for the counteraction of sicknesses in fish is viewed as another option and promising zone [2]. There is an extending interest in the utilization of medicinal herbs as immune stimulants in aquaculture [4], and the immunostimulant impacts of natural drugs in different fish species have been revealed [5]. In fisheries, immunostimulants enact the immune system of aquatic creatures and upgrade their ability for disease resistance [2].

Aquaculture is the highly dynamic and fastest-growing food-producing sector. The contribution of aquaculture to fish production is steadily increasing. The increasing demand for fish products and the commercialization of aquaculture led to the intensification of aquaculture. These conditions tend to adversely affect the health of the animals and produce a poor physiological environment and subsequently increase the susceptibility to infectious disease [6]. Diseases have become a significant constraint by causing mass mortality and severe economic loss. To overcome the disease problem, generally, antibiotics, chemotherapeutics, and disinfectants are used in aquatic systems, which in turn lead to drug resistance, human carry over, bioaccumulation, and pollution to the aquatic environment. Vaccination is also a useful prophylactic treatment, but due to its limited availability and pathogen-specific protective action, much attention has been diverted towards the application of immunostimulants [7].

*Aloe vera*, a spiky prickly plant like xerophyte, is a cluster framing lasting plant with a thick stringy root that produces enormous basal leaves, generally 12–16 for every plant, weighing up to 1.5 kg when fully grown. The plant develops when it is around 4 years of age and has a life expectancy of around 12 years. The leaves are long and opposite the base, tightening to a point with saw-like teeth along their edges. In a cross over the area, the plant shows a marginally curved appearance on the adaxial surface and an unmistakably raised appearance on the lower abaxial surface [8]. The leaves are covered with thick fingernail skin, underneath which epidermis and mesophylls are available, which is then separated into the upper chlorenchyma and lower parenchyma; as the rosettes develop, progressive leaves have less whitish spots and grey-greenish in color [9].

The medicinal plant *Aloe vera* has been known and used for centuries for its health, beauty, medicinal, and skincare properties [10, 11]. *Aloe vera* consists primarily of water and polysaccharides (pectin, cellulose, hemicellulose, glucomannan, acemannan, and mannose derivatives) and is composed of a long chain of acetylated mannose [12]. The biological activity of *Aloe vera*'s polysaccharides has been reported widely [13] and shown to act as an immunostimulant [14]. Acemannan has been reported to have antimicrobial properties, including antibacterial, antifungal, antiviral, and antiparasitic properties [15]. *Aloe vera* is recognized for its widespread use and reported healing powers [16], alleviating pain and treating a variety of ailments [17].

For millennia, residents have utilized the gel from *Aloe vera* leaves for mending and mellowing the skin. *Aloe vera* gel taken orally (by mouth) appears to help lower glucose levels in individuals with diabetes. It might likewise assist with bringing down cholesterol [18]. Common carp (*Cyprinus carpio*) is of the order Cypriniformes and the family Cyprinidae. It by and large occupies freshwater conditions, particularly lakes and streams, and seldom possesses saline water conditions [19].

The native carp is considered as vulnerable to extinction by the International Union for Conservation of Nature [20]. However, the species has also been domesticated and introduced in freshwater lakes and large rivers of Nepal, Asia, and worldwide. Recently, various mass mortalities have been reported in the Nepalese aquaculture system [21], and the use of immunostimulants is a suggestible alternative to antibiotics in this species.

There is restricted data accessible on the immunostimulatory, antitoxicity, and development impacts of *Aloe* in some fish species [22–25]. Zanuzzo et al. in the year 2017 clarified that dietary *Aloe vera* for 10 days before transport stress and infection with heat-killed *Aeromonas hydrophila* either improved or prevented the deficiency of innate immune activity in pacu (*Piaractus mesopotamicus*) after unpleasant taking care of and bacterial infection [26].

In another study, the *Aloe vera* methanolic extracts in the daily diet significantly improved the growth of red hybrid tilapia (*Oreochromis* sp.) [27]. Mesbah and Mohammadian in the year 2016 have explained that the oral administration of *Aloe vera* enhances non-specific immune responses in the shirbot (*Barbus grypus*) [28].

There was information in the literature on the role of dietary *Aloe vera*, in the counteraction of irresistible illnesses and histological changes to the skin and gastrointestinal tract in rainbow trout [29]. As far as we know, there is no data in the literature on the role of dietary *Aloe vera* on growth index, protein utilization, gastrosomatic index, etc., in common carp.

The present study aimed to investigate the effect of the *Aloe vera* extracts on growth index, protein utilization, gastrosomatic index, and gut histology in juvenile common carp *Cyprinus carpio* during intensive aquaculture.

## 2. Materials and Methods

### 2.1. Selection of Site

Affiliated to Tribhuvan University, Institute of Science and Technology, Amrit Campus, situated in the heart of Kathmandu Valley (Thamel), is one of the pure science campuses of the country. The aquaculture research lab (27.7172°N, 85.3240°E) of this campus is considered a Center of Excellence for Fisheries and Aquaculture Research; hence, this feeding trial was conducted to understand the feeding and growth pattern of carp concerning *Aloe vera* extracted diets.

### 2.2. Selection of Fish

Common carp (*Cyprinus carpio*) was selected for this experiment, and around one thousand juvenile carps with an average body weight of 1.73 ± 0.14 g were obtained from the hatchery of Janakpur (*a fish super-zone*), Nepal, to the Aquaculture Research Lab of Tribhuvan University, Amrit Campus, Kathmandu. Carps were acclimatized for three weeks in natural conditions prior to the beginning of the experiment. Water quality factors were recorded during the experiment as temperature 27 ± 13°C; pH 7.9 to 8.5, dissolved oxygen 5.8 to 6.5 ppm; NO_2_ < 0.1 mg/L, NH_3_ < 0.01 mg/L. The regular diet was fed during the acclimatization period (Table 1).

### 2.3. Preparation of *Aloe vera* Extract

Fresh *Aloe vera* plants, a spiky cactus-like xerophyte [8, 9], were collected from the local nursery within the campus, and the Department of Botany makes taxonomic identification. Briefly, 437 g of freshly cut leaves of *Aloe vera* was mixed with 100 ml of 70% ethanol and squeezed in a Whizzer, agitated on a shaker incubator for 12 hours, filtered through filter paper (Albert®, Pores 7–11, size 185 mm, England), and then lyophilized in a lyophilizer (Vacuum Gauge, USA). The extract for alcohol extract was 1.5%. The isolates were stored in tightly sealed dark containers in a freezer at −20°C for later use. Desiccations of the leaf determined the dry matter of *Aloe vera* at 50°C for 7 days. The humidity content of *Aloe vera* was 92%.

### 2.4. Preparation of Diets and Experiment Design

Altogether, four *Aloe vera* supplemented diets were prepared, as well as the diet without *Aloe vera*, controlled diet A (0.0%), while the other treated diets were as B, C, D, and E in which *Aloe vera* was supplemented as 0.2%, 0.4%, 0.8%, and 1.6%, respectively (Table 1). The proximate composition of a basal diet based on the formulation was prepared (Table 1). The experimental diets were prepared according to the method explained in the literature [7]. Other ingredients were used as per the standard norm of a laboratory. A total of 270 selected juvenile carp (1.73 ± 0.14 g) were circulated randomly at the rate of 15 fish per aquarium into 18 glass tanks (30” × 24” × 8”). Fish were fed twice daily (09 : 30 am and 3 : 30 pm) on their 3% of body weight. Sampling was on 30, 60, and 90 days, and during specific sampling; five fish were sampled from each tank and anesthetized using 0.03% tricaine methanesulfonate (MS-222) as per the guideline of the Canadian Council on Animal Care (CCAC 2005) [30] and then the fork length (cm) and body weight (g) of fish were recorded using a measuring tape and digital balance. For the gastrosomatic index (GaSI), three fish were sacrificed from each tank, and the gut of the fish were collected separately, weighed on 30, 60, and 90 days using the proper dissection procedure.

### 2.5. Examination Procedure

#### 2.5.1. Growth Performances

In this study, fish growth parameters were assessed in terms of percentage weight gain (PWG), specific growth ratio (SGR), food conversion ratio (FCR), feed efficiency rate (FER), protein efficiency ratio (PER), and condition factor (CF) calculated according to the following equations on 30th, 60th, and 90th days of feeding followed by percentage survival on regular monitoring. Gastrosomatic index (GaSI) was also monitored during the growth studies. All the fish weights were calculated in gram units.

PWG (g/fish) = (average final weight−average initial weight)/(initial weight) × 100.

SGR = (ln (final weight in grams)−ln (initial weight in grams)) × 100/*t* (in days).

FCR = food intake/weight gain.

FER = body weight gain/food intake.

PER = body weight gain/total protein intake.

CF = (body weight/(total length) 3) × 100.

Survival % = (number of survived fish/initial number of fish) × 100.

GaSI = (weight of gut/weight of body) × 100.

Here, the condition factor of fish is a parameter that is utilized broadly to get endurance, generation, development, and wellbeing of fish [31], and regularly, it tends to be utilized as a decent pointer of water quality or general strength of fish populaces that are occupying explicit natural surroundings or biological system [32]. To calculate CF (condition factor), the fork length (FL) of fish is estimated from the tip of the nose to the furthest limit of the center caudal-fin rays.

#### 2.5.2. Protein Profiles

Before sampling for tissue collection, all fish were starved for 24 h and then five fish per treatment were sampled and anesthetized with *tricaine methanesulfonate* (5 mg l^−1^) for 2–3 min, and then the liver tissues were collected through proper dissection weighed carefully. A 5% homogenate was prepared as per the method reported in the literature [7]. The total protein, albumin, globulin, and the ratio of albumin and globulin were estimated through Bio-Rad Bradford (1976) [33] protein assay reagents and available standard kits.

#### 2.5.3. Gut Histomorphology

The gut histomorphological examination was done at the end of the feeding trial on the 90th day and for it, the intestine was collected along with liver from the same anesthetized fish used during protein estimation. The proximal intestine was selected because it is where most of the nutrient absorption takes place [34]. During the process, the intestine tissue tests were fixed in 10% formaldehyde and handled by standard histological procedures (dried out in ethanol arrangement, inserted in paraffin, and sequentially segmented at 4 to 5 *μ*m) and stained with hematoxylin and eosin (H&E) [35]. Segments were assessed aimlessly under a light magnifying lens. Photos of the segments were made with a Nikon Eclipse 80i magnifying instrument (Nikon, Tokyo, Japan) furnished with a Nikon Digital Sight SD-MS camera and the Nikon programming NIS-Elements. Adobe Photoshop CS3 Extended was utilized for the last photographic readiness without adjusting the first uprightness of the photos. Morphometry consisted of measurements at 500x magnification of five randomly selected fields per intestinal tract.

### 2.6. Statistical Analysis

Analysis of data was done by utilizing a one-way analysis of variance (ANOVA) with *Aloe vera* consideration levels as a factor, utilizing the measurable bundle for the social sciences (SPSS) program (variant 22). Duncan's various reach tests at a critical degree of 95% were utilized to decide huge contrasts between treatments. The data introduced in the content, figures, and Table 1 implies means ± standard error, and *P* < 0.05 was considered as significant.

## 3. Results

### 3.1. Growth Performance

**Figure 1:**
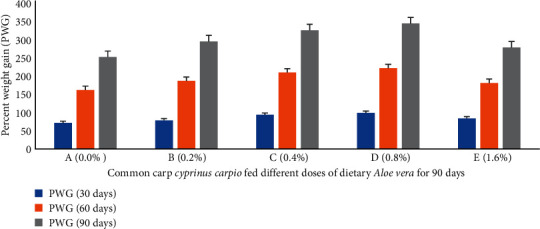
*Cyprinus carpio* fed varied doses of *Aloe vera* and percent weight gain (PWG) studied after 90 days of feeding trial.

**Figure 2:**
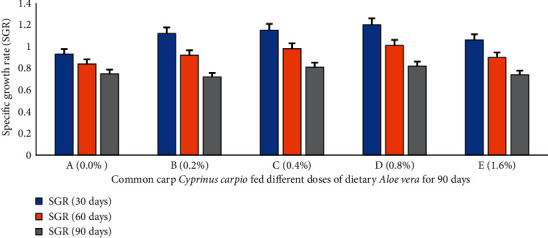
*Cyprinus carpio* fed varied doses of *Aloe vera* and specific growth rate (SGR) studied after 90 days of feeding trial.

**Figure 3:**
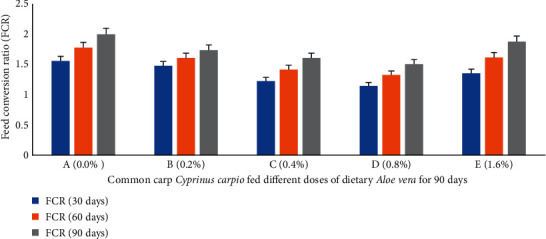
*Cyprinus carpio* fed varied doses of *Aloe vera* and feed conversion ratio (FCR) studied after 90 days of feeding trial.

**Figure 4:**
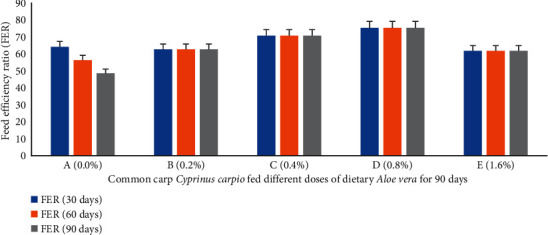
*Cyprinus carpio* fed varied doses of *Aloe vera* and feed efficiency ratio (FER) studied after 90 days of feeding trial.

**Figure 5:**
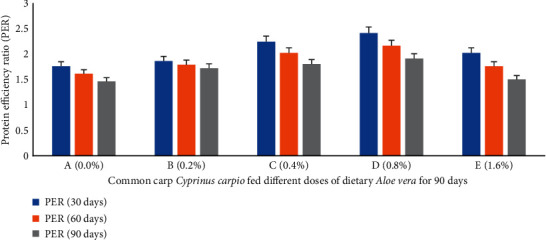
*Cyprinus carpio* fed varied doses of *Aloe vera* and protein efficiency ratio (PER) studied after 90 days of feeding trial.

**Figure 6:**
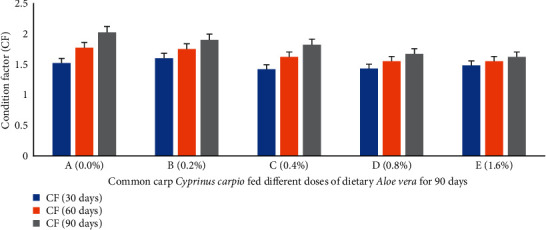
*Cyprinus carpio* fed varied doses of *Aloe vera* and condition factor (CF) studied after 90 days of feeding trial.

Percent weight gain (PWG) (Figure 1), specific growth rate (SGR) (Figure 2), feed conversion ratio (FCR) (Figure 3), feed efficiency ratio (FER) (Figure 4), protein efficiency ratio (PER) (Figure 5), and condition factor (CF) (Figure 6) of common carp after the 30^th^, 60^th^, and 90^th^ days of feeding on diets containing different levels of *Aloe vera* extracts experienced a significantly high (*P* < 0.05) growth performance among groups C (0.4%) and D (0.8%) compared to control A (0%) group.

**Figure 7:**
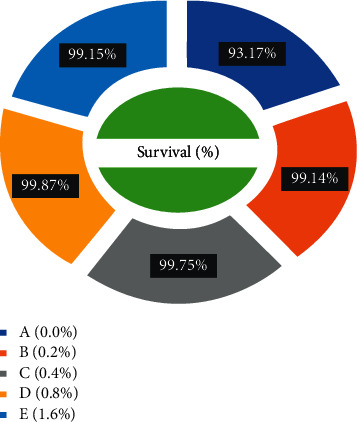
*Cyprinus carpio* fed varied doses of *Aloe vera* and survival percentage (S%) studied after 90 days of feeding trial.

The PWG and SGR of *C. carpio* were significantly affected in treated groups by dietary ethanolic *Aloe vera* extract (*P* < 0.05). Decreasing trends were observed in the FCR among the treated groups, and the lowest FCR was recorded in C (0.4%) diet-fed group (Figure 3). CF improved significantly in the treated groups and the lowest was found in the control diet-fed group. The survival percentage (S%) was higher among the treated groups (Figure 7).

**Figure 8:**
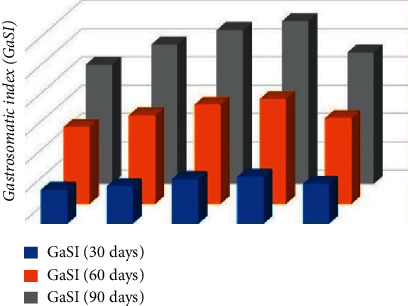
*Cyprinus carpio* fed varied doses of *Aloe vera* and Gastrosomatic index (GaSI) level studied after 90 days of feeding trial.

It was observed that as the dose of *Aloe vera* increased in the diet, the GaSI level also increased, and GaSI was significantly higher (*P* < 0.05) in common carp fed with D diet (0.8%) compared to control and other diet-fed groups (Figure 8). During the whole experimental period, the common carp fed with experimental diets tended to have better growth performance compared with the control.

### 3.2. Protein Profiles

**Figure 9:**
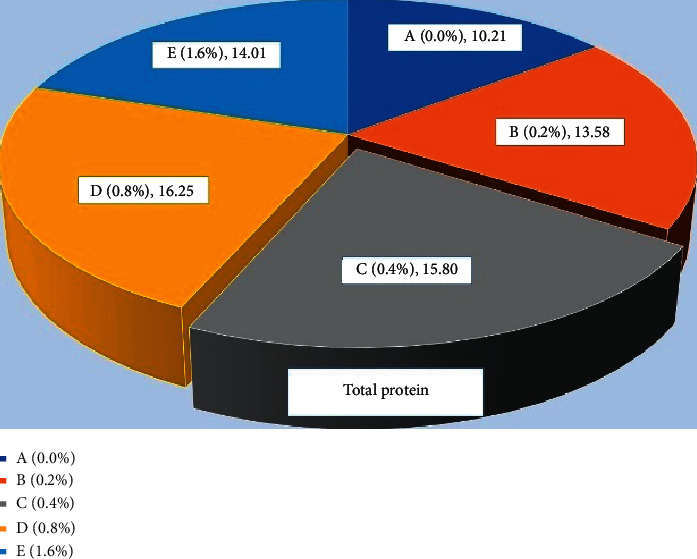
*Cyprinus carpio* fed varied doses of *Aloe vera* and total protein (TP) contents from liver tissues were studied after 90 days of feeding trial.

**Figure 10:**
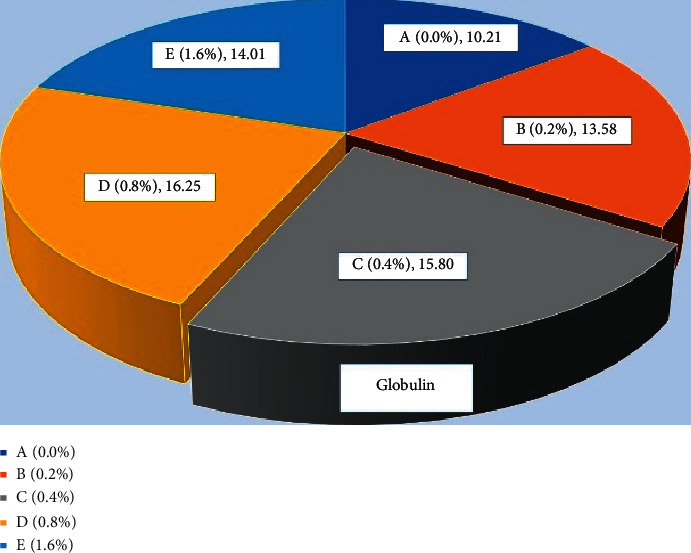
*Cyprinus carpio* fed varied doses of *Aloe vera* and globulin contents from liver tissues were studied after 90 days of feeding trial.

**Figure 11:**
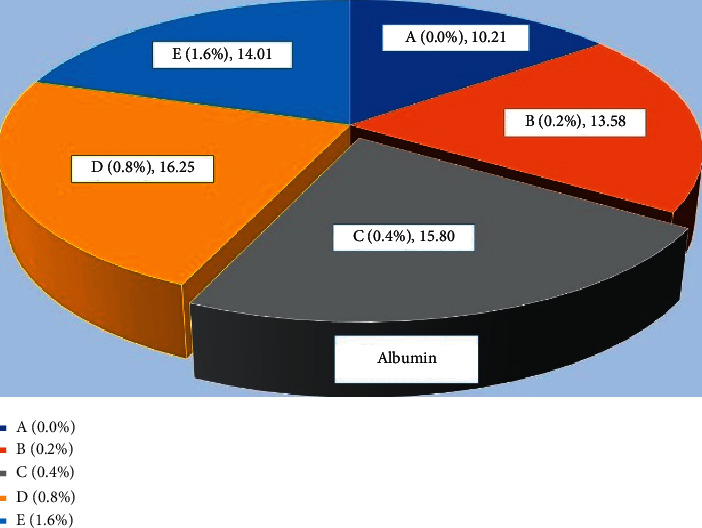
*Cyprinus carpio* fed varied doses of *Aloe vera* and albumin contents from liver tissues were studied after 90 days of feeding trial.

**Figure 12:**
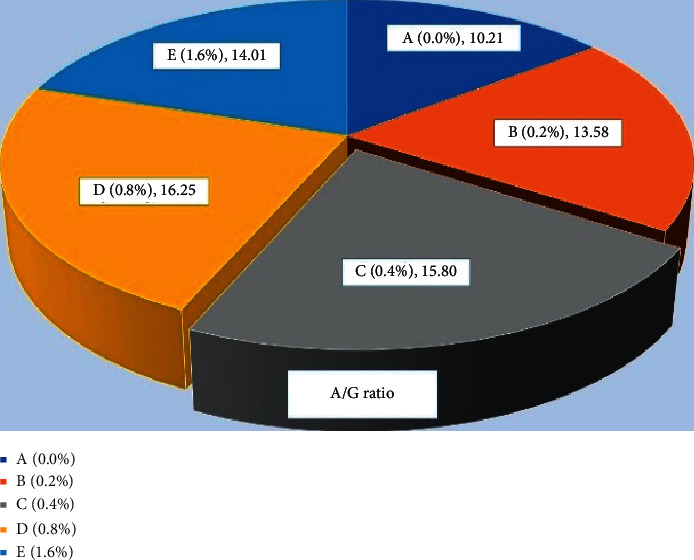
*Cyprinus carpio* fed varied doses of *Aloe vera* and ratio of albumin and globulin from liver tissues were studied after 90 days of feeding trial.

The total protein (Figure 9) level was found significantly high (*P* < 0.05) in fish fed with 0.4% *Aloe vera* in C group and 0.8% D group of carp as compared to other treated and control diet-fed groups. Similar results were observed in globulin (Figure 10) and albumin (Figure 11) of common carp fed with C (0.4%) diet of *Aloe vera* after 90 days of feeding trial. The highest ratio of albumin and globulin has been recorded in the D diet (0.8%) fed group compared to the control and another diet-fed group (Figure 12).

### 3.3. Gut Histomorphology

**Figure 13:**
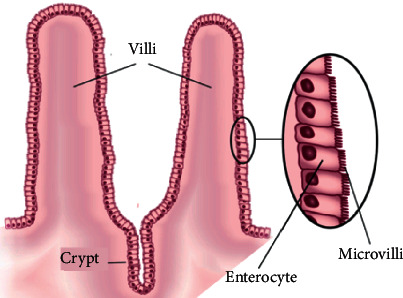
Ultrastructure of the small intestine [37].

The crypts of Lieberkühn (regularly referred to just as crypts) are pits between villi as in Figure 13. They are like the gastric pits in the stomach. The crypts contain stem cells that can create various diverse cell types, including enterocytes [36].

**Figure 14:**
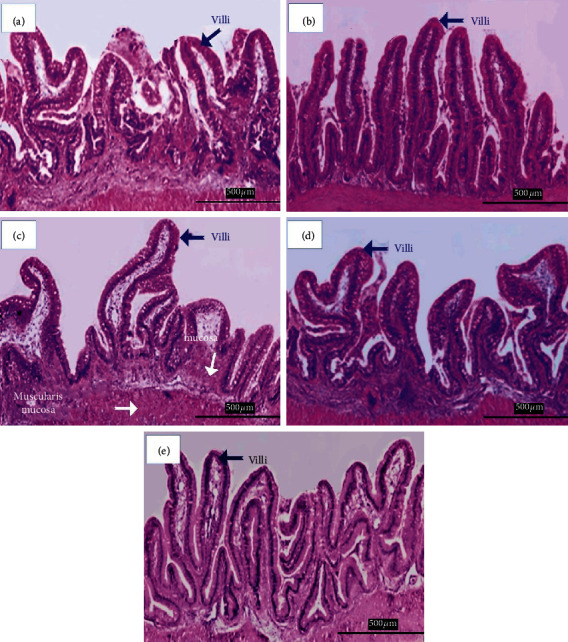
(a) *Cyprinus carpio* fed varied doses of *Aloe vera* and histology of distal intestine studied in control group A (0 mg/kg) diet-fed group after 90 days of feeding trial. (b) *Cyprinus carpio* fed varied doses of *Aloe vera* and histology of distal intestine studied in treatment group B (200 mg/kg) diet-fed group after 90 days of feeding trial. (c) *Cyprinus carpio* fed varied doses of *Aloe vera* and histology of distal intestine studied in treatment group C (400 mg/kg) diet-fed group after 90 days of feeding trial. (d) *Cyprinus carpio* fed varied doses of *Aloe vera* and histology of distal intestine studied in treatment group D (800 mg/kg) diet-fed group after 90 days of feeding trial. (e) *Cyprinus carpio* fed varied doses of *Aloe vera* and histology of distal intestine studied in treatment group E (1600 mg/kg) diet-fed group after 90 days of feeding trial.

After 90 days into the feeding trial, gut histology from all *Aloe vera* treated and control diet-fed carp was studied (Figures 14(a)–14(e)). Mucosa, muscular mucosa, and villi were spotted in the anterior gut of all groups, but the quality of villi was found better in diet-fed groups (Figures 14(b)–14(e)).

## 4. Discussion

The common carp *Cyprinus carpio* is a freshwater fish of lakes and rivers in Nepal and is believed to be in danger of extinction by the International Union for Conservation of Nature (IUCN), but its varieties have also been domesticated and distributed worldwide [38]. The omnivorous C*yprinus carpio* eats a herbivorous diet of aquatic plants and benthic worms [39]. The ethanol extracts of plants signify a continuous effort to find new compounds against pathogens, and about 20% of these submitted to the pharmacological or biological test [40]. A substantial number of new antibiotics available in the market are obtained from natural resources [41]. The medicinal plant *Aloe vera* is known for its healing properties for long years [42].

In the present study, the effects of crude extract of *Aloe vera* on percent survival, growth condition factors, protein profile, gastrosomatic index, and gut histomorphology were studied for 90 days of common carp fed *Aloe vera* in laboratory conditions. Plants like onion, garlic, and black cumin seed have been tested for growth-promoting activities, feed conversion, and expansion of protein digestibility in aquatic animals [43]. The present study showed a significantly higher percentage (99.87%) of survival in the D diet-fed group compared to the control (93.17%). The results for growth performance in percentage weight gain (PWG) and specific growth ratio (SGR) in common carp fed groups *Aloe vera* supplemented with 0.4% and 0.8% of C and D groups look much better than the control group A. The present results are in harmony with the literature [44]. The latter reported 0.5% and 2.5% ethanolic dietary *A. vera* inclusion levels and they were able to improve growth performance significantly in common carp. Heidarieh et al. in the year 2013 revealed 0.1% and 1% *Aloe vera* inclusion level increased growth performance in rainbow trout (*O. mykiss*) [29] and significant 3% growth performance (GP) found in percent weight gain (PWG), feed conversion efficiency (FCE), protein efficiency ratio (PER), and specific growth rate (SGR) of *Nile tilapia* explained by Shalaby et al. in the year 2006 [45]. The present results are also in agreement with the literature [46]. Khattab et al. [46] found that the diet of Biogen increased feed intake and improved feed conversion ratio (FCR), and protein efficiency ratio (PER) in Pangasius.

In this experiment, total protein, globulin, and albumin have been quantified from the liver tissues of treated and control diet-fed carp. It was surprising that carp fed with *Aloe vera* supplemented diet perform drastically higher (*P* < 0.05) in all the treated groups compared to the control diet-fed group A. Similar results were observed in globulin and albumin levels. The total protein, globulin, and albumin levels were higher in *Aloe vera* 0.4% and 0.8% diet-fed group. The growth and immune response of silver striped catfish *(Pangasianodon hypophthalmus)* took care of lapsi fruits *(Choerospondias axillaris)* and discovered that absolute protein, albumin, and globulin level was higher in fish taking care of 0.4% lapsi fruits diet [7]. In agreement with the present findings, the use of *Allium sativum* and *Zingiber officinale* increased total protein concentration significantly (*P* < 0.05) and concluded that they protect the liver against harmful agents and free radical-mediated toxic damages to the liver cells explained by Ajeel and Al-Faragiin 2013 [47]. Similar results were also found in the literature [48], and Hamza et al. in the year 2008 [49] assessed the protein expression profile in the liver of 34-day-old Pikeperch larvae fed with three isotropic and isolipidic formulated diets. Hassan and Javed in the year 2000 [50] also found that a higher concentration of sarcoplasmic protein in *Catla catla* corresponded to a decrease of myofibrillar protein. By adding an extract of ginger to fish, the diet improved the protein level; the highest protein was observed in carp fed with 1% ginger extract [51]. The relationship between the weight of the alimentary canal and the weight of fish helps in determining the feeding condition in different months and seasons denoted by gastrosomatic index, and GaSI of common carp in the present study was found higher on the 30th, 60th, and 90th days of *Aloe vera* treated diets compared to control diet-fed group. The highest value, 5.22 **±** 0.04, of the gastrosomatic index (GaSI) was recorded in *Acanthopagrus latus* by Sourinejad et al. [52] and Pramanik and Mohanty [53] recorded the highest 8.1% GaSI value of *Rutilus crisis* collected from Iranian waters.

Histological monitoring of the structure of the alimentary canal in carp fed new ingredients provides essential information [54], but so many studies have not been carried out with carp [55].

After 90 days into the feeding trial, gut histology from all the *Aloe vera* treated and control diet-fed carp found villi spotted in the anterior gut of all groups. Such changes are because of *Aloe vera* present in the tissues.

The taller, narrower, and routinely molded villi and the higher number of villi per unit zone are pointers that the capacity of the intestinal villi is initiated [56, 57]. In general, these villi gave a more prominent surface area for absorption of accessible supplements. These realities recommend that the villus capacity may be initiated after taking care of dietary *Aloe vera* in rainbow trout [29].

Similarly, morphological changes in the distal intestine are responsible for the growth that was observed in response to *Aloe vera* supplemented diets [58]. Moreover, we can compare our findings with the literature [59] that *Ziziphus mauritiana* leaf powder (ZLP) was used to see the growth performance, intestinal histomorphology, and growth-related gene expression of *Nile tilapia* (*Oreochromis niloticus*). The stomach demonstrated an expansion in the thickness of the gastric mucosa and muscularis in totally treated gatherings with the most elevated qualities in 10 g/kg took care of gathering. The intestinal mucosal folds, statures, widths, zone, and edge of the villi and thickness of strong layers were fundamentally (*P* < 0.05) higher in gatherings took care of with ZLP, which was affirmed by utilizing scanning electron microscopy. In this manner, ZLP can be utilized viably in tilapia consuming fewer calories for improving the development and intestinal wellbeing. These days, the use of bioactive compounds through herbal supplementation in the aquafeed is growing interest for scientists and aquafarmers. Therefore, the application of *Aloe vera* is very common in other animals but it is a new attraction from the aquaculture perspective.

## 5. Conclusion

The results of *Aloe vera* studies for other medical conditions have been less clear. This paramount attitude of this research was to understand the effects of *Aloe vera* on the growth and protein profiles of carp, and in the results, we can observe that *Aloe vera* extract is useful for fish growth by enhancing protein contents in the liver tissues. Survival and growth performance followed by gastrosomatic index and condition factors studied indicate the use of *Aloe vera* in aquaculture carp growth. In contrast, the protein profile and the ratio of albumin and globulin indicate the immunity level in carp fed *Aloe vera* supplemented diets. Gut histomorphology study is further proof, and the performance of the growth of villi in the treated group, especially in C (0.4%) and D (0.8%) diet-fed group, boosts the morphophysiology of carp compared to the control A (0%) group. Finally, this experiment is an initial trial using *Aloe vera* extract supplemented diets in the Nepalese aquaculture system, which inspires new researchers to work further using the medicinal herb in aquaculture as an alternate dose of vaccine for carp growth. From the economic point of view, it solves food security for aquafarmers.

## Figures and Tables

**Table 1:** Percentage of the experimental diets containing supplement of different levels of *Aloe vera* (gel extract) and proximate composition.

Ingredients	Experimental diets (% inclusion)				
	A (0%)	B (0.2%)	C (0.4%)	D (0.8%)	E (1.60%)
Fish meal†	29.31	29.31	29.31	29.31	29.31
Soya meal^‡^	13.16	13.16	13.16	13.16	13.16
Groundnut oil cake†	9.17	9.17	9.17	9.17	9.17
Rice powder†	15.52	15.32	15.12	14.72	13.92
Wheat flour†	14.43	14.43	14.43	14.43	14.43
Corn flour†	11.37	11.37	11.37	11.37	11.37
Sunflower oil†	3	3	3	3	3
Cod liver oil^†^	2	2	2	2	2
Vitamin and mineral premix^§^	1	1	1	1	1
Betaine hydrochloride††	0.02	0.02	0.02	0.02	0.02
BHT (butylated hydroxytoluene)††	0.02	0.02	0.02	0.02	0.02
*Aloe vera* (*Aloe barbadensis*) extract	0	0.2	0.4	0.8	1.6
CMC (carboxymethylcellulose)††	1	1	1	1	1

Total	100	100	100	100	100

Proximate analyses					
Protein, (%)	48.25	—	—	—	—
Fat, (%)	18.86	—	—	—	—
Moisture, (%)	9.36	—	—	—	—
Ash, (%)	12.25	—	—	—	—
Carbohydrate (%)	8.23	—	—	—	—
Energy (kJ·kg ^−1^)	2025	—	—	—	—

^†^Ingredients such as fish meal, soya meal, groundnut oil cake, rice powder, wheat flour, cornflour, sunflower oil, and cod liver oil were procured from the local market of Kathmandu Valley. ^‡^Ruchi Soya Industries, Raigad, India. ^§^Composition of vitamin-mineral mix (EMIX PLUS) (quantity 2.5 kg ^−1^). Vitamin A: 55,00,000 IU; vitamin D3: 11,00,000 IU; vitamin B2: 2,000 mg; vitamin E: 750 mg; vitamin K: 1,000 mg; vitamin B6: 1,000 mg; vitamin B12: 6 *µ*g; calcium pantothenate: 2,500 mg; nicotinamide: 10 g; choline chloride: 150 g; Mn: 27,000 mg; I: 1,000 mg; Fe: 7,500 mg; Zn: 5,000 mg; Cu: 2,000 mg; Co: 450 mg; Ca: 500 g; P: 300 g; L-lysine: 10 g; DL-methionine: 10 g; selenium: 50 mgl^−1^; Satwari: 250 mgl^−1^ (*Lactobacillus,* 120 million units, and yeast culture, 3000 crore units). ^††^Himedia Laboratories, Mumbai, India.

## Data Availability

The data used to support the findings of this study are included within the article.
